# Substance P Improves Renal Ischemia Reperfusion Injury Through Modulating Immune Response

**DOI:** 10.3389/fimmu.2020.00600

**Published:** 2020-04-23

**Authors:** Dong-Jin Kim, Ju-Young Moon, Su-Mi Kim, Jung-Woo Seo, Yu Ho Lee, Su Woong Jung, Kipyo Kim, Yang Gyun Kim, Sung-Jig Lim, Sangju Lee, Youngsook Son, Sang-Ho Lee

**Affiliations:** ^1^Division of Nephrology, Department of Internal Medicine, Kyung Hee University Hospital at Gangdong, Kyung Hee University, Seoul, South Korea; ^2^Laboratory of Tissue Engineering, Department of Genetic Engineering, College of Life Science and Graduate School of Biotechnology, Kyung Hee University Global Campus, Yongin, South Korea; ^3^Division of Nephrology, Department of Internal Medicine, CHA Bundang Medical Center, CHA University, Seongnam, South Korea; ^4^Division of Nephrology and Hypertension, Department of Internal Medicine, College of Medicine, Inha University, Incheon, South Korea; ^5^Department of Pathology, Kyung Hee University Hospital at Gangdong, Kyung Hee University, Seoul, South Korea; ^6^Lee Sangju Clinic, Daejeon, South Korea

**Keywords:** fibrosis, immune modulation, ischemia reperfusion injury, macrophage, substance P

## Abstract

Substance P (SP), an injury-inducible messenger that mobilizes bone marrow stem cells and modulates the immune response, has been suggested as a novel target for therapeutic agents. We evaluated the role of SP as an immune cell modulator during the progression of renal ischemic/reperfusion injury (IRI). Unilateral IRI induced the transient expression of endogenous SP and the infiltration of CCR7^+^ M1 macrophages in injured kidneys. However, SP altered the intrarenal macrophage polarization from CCR7^+^ M1 macrophages to CD206^+^ M2 macrophages in injured kidneys. SP also modulated bone marrow-derived neutrophils and mesenchymal stromal cells after IRI. SP treatment for 4 weeks starting one week after unilateral IRI significantly preserved kidney size and length and normal tubular structures and alleviated necrotic tubules, inflammation, apoptosis, and tubulointerstitial fibrosis. The beneficial effects of SP were accompanied by attenuation of intrarenal recruitment of CD4, CD8, and CD20 cells and abnormal angiogenesis. The immunomodulatory effect of SP suggested that SP could be a promising therapeutic target for preventing the progression of acute kidney injury to chronic kidney disease.

## Introduction

Despite recent advances in intensive care management, severe and dialysis-dependent acute kidney injury (AKI) occurs in more than 5% of critically ill patients and is associated with high mortality (1). AKI is also an important risk factor for progression to chronic kidney disease (CKD), particularly in elderly patients (2, 3). After AKI, renal cell mass is reduced by necrosis and apoptosis and subsequently progresses to fibrosis without undergoing regeneration, and this process is directly related to the development of CKD (4, 5). Immune cell infiltration after injury and the subsequent development of inflammation are related to the critical progression of AKI to CKD (6, 7).

Immunomodulation by medications or bone marrow-derived mesenchymal stromal cells (MSCs) has been suggested as a potential treatment for AKI (8, 9). Recent studies have demonstrated that the major roles of stem cells in AKI involve both paracrine and endocrine effects and are associated with trophic, anti-inflammatory, and immunomodulatory mechanisms instead of differentiation to kidney cells (10, 11). However, because most of these experiments are performed using external MSCs, there are risks such as tumorigenicity and immunogenicity, which are well-known side effects of stem cell therapy (12). Another issue of stem cell therapy is that although some reports of improved immune-related cytokines have been published, few studies have examined how to modulate immune cell activation after AKI.

Substance P (SP) is an 11-amino acid neuropeptide that is involved in pain perception by activating neurokinin 1 (NK-1) receptors (13). SP is also widely expressed in non-neuronal tissues, including the kidney (13). Hong et al. demonstrated that SP acts not only as a messenger that indicates injury but also as a mobilizer of CD29^+^ stromal-like cells, which are produced in the bone marrow and participate in wound healing (14). Recent studies have shown that SP has an anti-inflammatory effect by inhibiting tumor necrosis factor-alpha (TNF-α) and inducing IL-10 (15–17). In the case of ischemia reperfusion injury (IRI), SP improves the injury by increasing MSC and endothelial progenitor cell (EPC) in the blood circulation and reducing TNF-α (18). Furthermore, SP directly participates in the transition between M1 macrophages and M2 macrophages (19). However, SP expression has not been investigated in kidney disease, and whether SP has a beneficial effect on AKI is unclear.

In the present study, we hypothesized that SP could attenuate AKI by modulating immune cells and would prevent AKI to CKD progression. We administered SP to a renal IRI model and evaluated the effect on immune modulation and bone marrow-derived cells and chronic progression after AKI.

## Methods

### Animals and Experimental Design

A unilateral renal IRI model was established using 8-week-old male C57BL/6 mice. Mice were anesthetized with a combination of tiletamine–zolazepam (45 mg/kg) and xylazine (10 mg/kg). After incising the left back of mice, we induced ischemia in the left kidneys of the mice using a microclamp to obstruct blood circulation for 45 min. During the ischemic period, the body temperature of the mice was maintained at 36–37°C using a heating pad and a rectal temperature probe.

1. To identify the relationship between IRI and SP, we induced IRI in C57BL/6 mice and sacrificed at 1, 3, and 7 days after IRI. The mice were divided into sham and IRI groups (*n* = 6).
2. To evaluate the effects of IRI on mobilization and homing of bone marrow-derived cells to injured kidneys, we induced IRI in C57BL/6 mice and GFP-positive bone marrow-transplanted mice and sacrificed the mice at 1 and 3 days after IRI. The mice were divided into the sham, IRI + saline and IRI + SP groups (*n* = 6 per group), and SP (Calbiochem, MA, USA) was administered twice via the tail vein 1 day before IRI and immediately after reperfusion.
3. To demonstrate the effect of SP on AKI to CKD progression, we induced IRI in C57BL/6 mice and GFP-positive bone marrow-transplanted mice. After 1 week, SP (Calbiochem, MA, USA), the NK-1 receptor antagonist RP67580 (Tocris, Bristol, UK), or saline was administered via the tail vein twice per week for 4 weeks. Urine was collected from individual metabolic cages for 24 h at 0, 1, and 2 weeks after IRI. Kidney samples were obtained at 1, 3, and 5 weeks after IRI. The level of neutrophil gelatinase-associated lipocalin (NGAL) in the urine was quantified using ELISAs (R&D, Minnesota, USA).

All animal experiments were performed in compliance with the guidelines of the Animal Research Ethics Committee of Kyung Hee University and Institutional Animal Care and Use committee Kyung Hee University Hospital at Gangdong, Seoul, Korea (approval number: KHNMC AP 2016-007).

### Bone Marrow Transplantation

C57BL/6 mice received 10 Gy of total body irradiation from an X-ray source. Bone marrow cells (5 × 10^6^) were obtained from C57BL/6-tg (CAG-EGFP) mice (Japan SLC Inc., Shizuoka, Japan) and then transplanted via the tail vein into irradiated C57BL/6 mice. After 4 weeks, we assessed the blood, bone marrow, spleen and kidney through FACS analysis to identify the GFP^+^ bone marrow transplant chimeric mice.

### Histology

Sections were cut at a thickness of 4 μm. For the histological assessment of tubulointerstitial injury (tubular necrosis, tubular atrophy, and glomerular cysts), the sections were stained with periodic acid-Schiff reagent. Ten corticomedullary fields were examined in each section at 200x magnification, and a semiquantitative analysis of tubulointerstitial injury was performed. The total tubular injury was graded on a scale of 0 to 5 based on the percentage of normal tubules and the amount of tubular necrosis and tubular atrophy as follows: 0, absent; 1, 1–25%; 2, 26–50%; 3, 51–75%; 4, 76–99%; and 5, 100%. Fibrosis was quantified using Masson's trichrome staining and computer-assisted image analysis. Apoptosis was quantified using an *In Situ* Cell Death Detection kit (Roche, IN, USA) according to the manufacturer's protocol. To quantify cell proliferation, we performed PCNA (Serotec, North Carolina, USA) staining according to the manufacturer's protocol. PCNA^+^ cells were counted in ten corticomedullary fields from each section at 200x magnification. For immunohistochemistry, we used the Bond Polymer Refine Detection system (Vision BioSystems, Australia) with antibodies against SP, CD4, CD8a, and CD20. Four-micron-thick kidney sections were deparaffinized using Bond Dewax solution, and an antigen retrieval procedure was then performed using Bond ER solution for 30 min at 100°C. Endogenous peroxidase activity was quenched by incubating the tissues with hydrogen peroxide for 5 min. The sections were incubated with SP (1:50; Santa Cruz, CA, USA), CD4 (1:100; Abcam, Cambridge, UK), CD8a, and CD20 (1:100; Abcam, Cambridge, UK) antibodies using a biotin-free polymeric horseradish peroxidase-linked antibody conjugate system (Vision BioSystems, USA). To assess T and B lymphocyte infiltration, we counted CD4^+^/CD8a^+^/CD20^+^ cells in 6 randomly selected fields from each section at 200x magnification under a light microscope. The intensity of SP immunostaining was analyzed using ImageJ software (National Institutes of Health, Bethesda, MD).

### Immunofluorescence

Kidneys from GFP^+^ bone marrow transplant chimeric mice were embedded in optimal cutting temperature compound (OCT compound; Sakura Finetec, Tokyo, Japan). Sections were cut at a thickness of 7 μm. The sections were incubated with CD45 (1:100; Thermo Fisher Scientific, Waltham, MA, USA) antibody and then incubated with Rat DyLight® 594 (1:200; Bethyl Laboratories, Montgomery, TX, USA). To assess GFP^+^ bone marrow-derived cells and CD45^+^ immune cells, we randomly selected ten fields from each section at 400x magnification under a confocal microscope (LSM 700; Carl Zeiss Microscopy GmbH, Germany), and the intensity of GFP (green) and CD45 (red) was analyzed using ImageJ software (National Institutes of Health, Bethesda, MD).

### FACS Analysis

Kidneys were placed into Stomacher bags containing 10 ml of RPMI buffer and ground using a Stomacher 80 Biomaster (Seward Lab Systems). The samples were centrifuged at 1,800 rpm for 10 min, and then, the cell pellet was resuspended in 40% Percoll (GE Healthcare, Sweden), placed on top of a layer of 80% Percoll and centrifuged at 3,000 rpm for 30 min (stopped without using the break braking). The cell layer located the middle of tube was collected and PBS was added for washing. The cell pellet was then resuspended in FACS buffer. The following antibodies were used for FACS: CCR7-Alexa 488, CD206-PE-Dazzle 594, CD133-PE, Ly6G-Cy7, CD68-FITC, CD86-PerCP/Cy5.5, CD169-Cy7 (Biolegend, CA, USA), CD163-PE (Invitrogen, CA, USA), CD11b-PE, CD309-PE, CD309-APC, CD31-FITC, CD29-FITC, CD105-APC, CD45-PE, CD45-APC, and Sca-1-PE (Miltenyi Bio, CA, USA). The cells were analyzed using a FACSCalibur flow cytometry system (BD Biosciences, CA, USA) and Cytomics FC 500 (Beckman Coulter, CA, USA), and data analysis was performed using FlowJo software version 10 (TreeStar, OR, USA).

### Isolation of Total RNA and Real-Time PCR

Total RNA was extracted from kidney tissue using a Total RNA Isolation Kit (MACHEREY-NAGEL, NY, USA). Complementary DNA was synthesized using random primers (Promega, WI, USA) and M-MLV reverse transcriptase (Mbiotech Inc., South Korea). Real-time PCR was performed in reactions with a final volume of 20 μl that contained 1 μl of cDNA, 10 pmol of each sense and antisense primer, and 17 μl of SYBR Green PCR Master Mix (FastStart Universal SYBR Green Master; Roche, IN, USA). The primer sequences used for TAC1, TGF-β1, CTGF, collagen IV-a1, collagen I-a1, angiopoietin-1, angiopoietin-2, vascular endothelial growth factor A (VEGF-A), VEGF receptor 2 (VEGFR2), IFN-γ, IL-1β, TNF-α, IL-17a, and 18S are shown in Supplementary Table 1. Each sample was run in duplicate in separate tubes to quantify target gene expression, and the results were normalized to 18S expression.

### Western Blot Analysis

We lysed kidney tissues with protein extraction solution (PRO-PREP™; iNtRON Biotechnology, INC., Sungnam, Korea). After separation with 15% SDS-PAGE and electrotransfer of the proteins to a PVDF membrane(Millipore, Madrid, Spain), we blocked the membrane for 1 h at room temperature. And then the membrane was incubated overnight with antibodies to SP (1:500; Santa Cruz Biotechnology, CA, USA) and β-actin (1:2000; Santa Cruz Biotechnology, CA, USA) at 4°C. The membranes were subsequently stained with horseradish peroxidase-conjugated goat anti-rabbit or mouse immunoglobulin G (1:5,000, Santa Cruz Biotechnology, CA, USA). The immunoreactive bands were detected by chemiluminescence (enhanced chemiluminescence; BioFX Laboratories Inc., MD, USA). β-actin was used as an internal control.

### Statistical Analysis

The data are expressed as the mean ± SEMs. Comparison between contralateral and IRI kidneys was made with paired *t*-test, followed by the Wilcoxon test. Comparison about time point was made with unpaired *t*-test, followed by Mann-Whitney test. Comparison between groups was made with one-way ANOVA, followed by the Kruskal-Wallis nonparametric analysis. *P* < 0.05 were considered statistically significant. All of the analyses were completed using Graphpad Prism 8 Software (GraphPad Software Inc., CA, USA) for Windows.

## Results

### IRI Induced Endogenous Renal SP Expression

**Figure 1:**
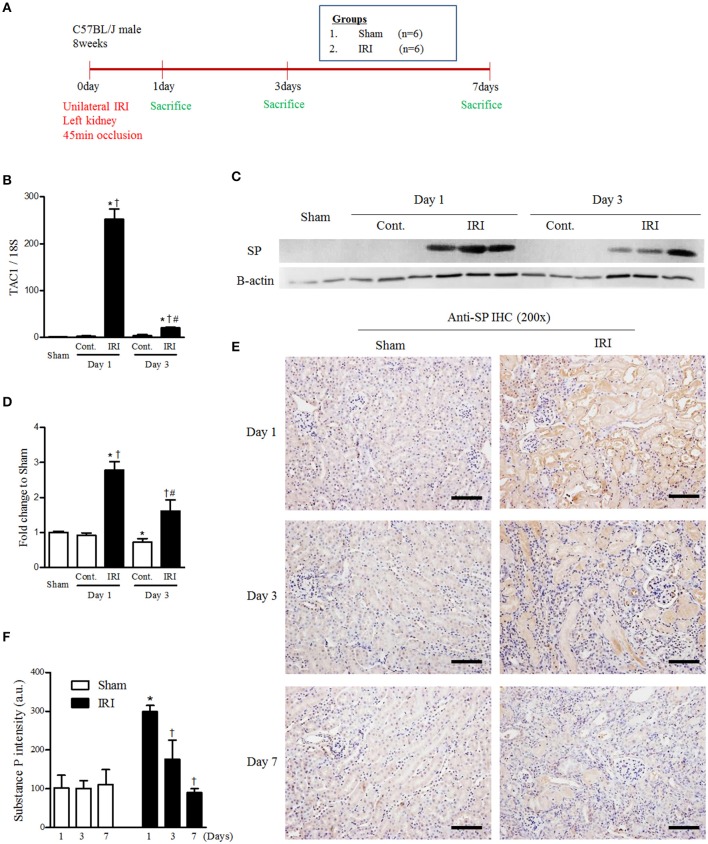
IRI induced endogenous renal SP expression. **(A)** Experimental design. Unilateral IRI was induced in the left kidneys of C57BL6 mice and sacrificed at 1, 3, and 7days after IRI. For these experiments, the mice were divided into sham (*n* = 6) and IRI (*n* = 6) groups. **(B)** The mRNA expression levels of TAC1 at 1 and 3 days after IRI. **(C)** The protein expression of endogenous SP at 1 and 3 days after IRI. **(D)** The graph of SP protein expression. **(E)** Immunohistochemical detection of SP in the kidneys at 1, 3, and 7 days after reperfusion. **(F)** The intensity of the SP^+^ fraction. Values are shown as the mean ± S.E.M. ^*^p < 0.05 vs. the sham group, ^†^*p* < 0.05 vs. the contralateral kidney. ^#^*p* < 0.05 vs. at 1 day after IRI. Scale bar = 80 μm.

To determine whether endogenous SP expression is induced by IRI, we induced IRI and sacrifice at 1, 3 and 7 days after IRI (Figure 1A). We performed qRT-PCR to measure the mRNA expression change of TAC-1, the precursor of SP, in sham, contralateral and IRI kidneys at 1 and 3 days after IRI. The mRNA level of TAC-1 was highly induced in IRI kidneys 1 day after IRI. However, TAC-1 mRNA expression was transiently increased only at the site of injury at the beginning of the injury (Figure 1B and Supplementary Figure 1). The protein expression of endogenous SP also increased and peaked in IRI kidneys 1 day after IRI (Figures 1C,D). Endogenous SP expression was substantially higher in most of the tubules in the IRI kidneys than in normal kidney tubules at 1 day after IRI. However, the tubular expression of SP gradually decreased, returning to baseline levels at 7 days after reperfusion (Figures 1E,F). These results confirmed that SP acts as a key rapid responder to stressors in the kidney and that IRI induces a significant but transient increase in the renal expression of SP.

### SP Modulated IRI-Induced Macrophage Polarization

**Figure 2:**
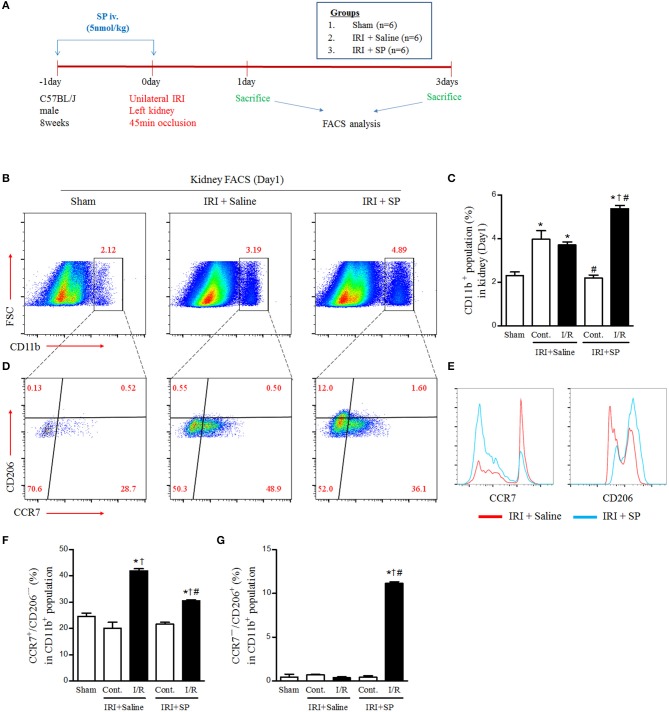
SP modulated IRI-induced macrophage polarization. **(A)** Experimental design. Unilateral IRI was induced in the left kidney in C57BL6 mice. For these experiments, the mice were divided into the following three groups: normal control (sham, *n* = 6), saline-treated after IRI (IRI + saline, *n* = 6) and SP-treated after IRI (IRI + SP, *n* = 6). Saline or SP was administered to mice at 1 day before IRI and immediately after reperfusion. The mice were sacrificed at 1 day after IRI. **(B)** The intrarenal CD11b^+^ cell populations. **(C)** The percentage of intrarenal CD11b^+^ cell populations. **(D)** The intrarenal CCR7^+^/CD206^−^ M1 macrophage and CCR7^−^/CD206^+^ M2 macrophage populations in CD11b^+^ cell populations. **(E)** The histogram of CCL7 and CD206 in CD11b^+^ cell populations. **(F)** The percentage of intrarenal CCR7^+^/CD206^−^ M1 macrophages in CD11b^+^ cell populations. **(G)** The percentage of intrarenal CCL7^−^/CD206^+^ M2 macrophages in CD11b^+^ cell populations. Values are shown as the mean ± S.E.M. ^*^p < 0.05 vs. the sham group, ^†^*p* < 0.05 vs. the contralateral kidney, ^#^*p* < 0.05 vs. the IRI + saline group.

Macrophage infiltration and polarization after IRI have pivotal roles in early injury progression and healing. We administered SP or normal saline one day before and right after IRI to evaluate the effects of SP on the polarization of macrophages after IRI and then performed FACS analysis (Figure 2A). At 1 day after IRI, the population of CD11b-positive cells was increased in the contralateral and injured kidneys. Interestingly, SP increased the population of CD11b-positive cells in the injured kidney rather than the contralateral kidney (Figures 2B,C). In the CD11b-positive population, renal CCR7^+^/CD206^−^ M1 macrophages were increased by IRI. However, these renal CCR7^+^/CD206^−^ M1 macrophages were decreased and CCR7^−^/CD206^+^ M2 macrophages were increased by SP treatment compared to normal saline (Figures 2D–G). The population of CD68-positive cells also was increased in the contralateral and injured kidneys and the increase was higher in injured kidneys than in contralateral kidneys. Although there was no significant difference in the population of CD68-positive cells by SP, the population of CD163-positive cells was increased by SP (Supplementary Figure 2). In the CD68-positive population, renal CD68^+^/CD86^+^ cells and CD68^+^/CD169^+^ cells were increased by IRI. However, both populations were decreased by SP treatment (Supplementary Figure 3). These results demonstrated that SP modulates the early innate immune response after IRI by inducing M2 polarization.

### SP Modulated Bone Marrow-Derived Neutrophil Infiltration and MSCs After IRI

**Figure 3:**
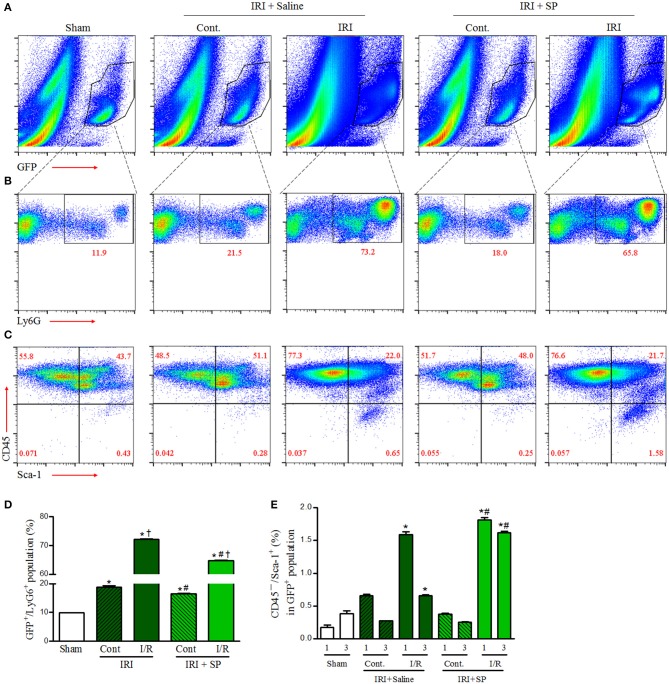
SP modulated bone marrow-derived neutrophil infiltration and MSCs after IRI. Unilateral IRI was induced in the left kidney in GFP-positive bone marrow-transplanted mice. For these experiments, the mice were divided into the following three groups: normal control (sham, *n* = 6), saline-treated after IRI (IRI + saline, *n* = 8) and SP-treated after IRI (IRI + SP, *n* = 8). Saline or SP was administered to the mice at 1 day before IRI and immediately after reperfusion. The mice were sacrificed at 1 and3 days after IRI. **(A)** The intrarenal bone marrow-derived GFP^+^ cell populations at 3 days after IRI. **(B)** The intrarenal GFP^+^/Ly6G^+^ cell populations at 3 days after IRI. **(C)** The intrarenal CD45^−^/Sca-1^+^ cells in bone marrow-derived GFP^+^ cell populations at 3 days after IRI. **(D)** The percentage of the intrarenal GFP^+^/Ly6G^+^ cell populations at 3 days after IRI. **(E)** The percentage of intrarenal CD45^−^/Sca-1^+^ cells in bone marrow-derived GFP^+^ cell populations at 1 and 3 days after IRI. Values are shown as the mean ± S.E.M. ^*^p < 0.05 vs. the sham group, ^†^*p* < 0.05 vs. the contralateral kidney, ^#^*p* < 0.05 vs. the IRI + saline group.

Bone marrow-derived cell infiltration after kidney injury has both harmful effects via activation of inflammation and beneficial effects through promotion of regeneration. To evaluate the effects of SP on the infiltration of bone marrow-derived inflammatory cells and mesenchymal stem cells to the kidney after IRI, we performed FACS analysis of the kidneys of GFP^+^ bone marrow transplant chimeric C57BL6 mice after IRI. The bone marrow-derived GFP^+^ cell population was increased at 1 and 3 days after IRI in both the contralateral and IRI kidneys (Figure 3A). SP alleviated the increase in renal GFP^+^/Ly6G^+^ neutrophil infiltration at 3 days after IRI compared to that of the saline-treated group (Figures 3B,D). Intrarenal GFP^+^/CD45^−^/Sca-1^+^ MSCs were increased one day after IRI, but these populations were normalized to the baseline on day 3 in saline-treated mice. However, SP led to the maintenance of the intrarenal bone marrow-derived GFP^+^/CD45^−^/Sca-1^+^ MSC population until 3 days after IRI (Figures 3C,E). These results demonstrated that SP could improve inflammation and regeneration after IRI by modulating bone marrow-derived cells.

### SP Attenuated Progressive Renal Injury and Fibrosis After AKI

**Figure 4:**
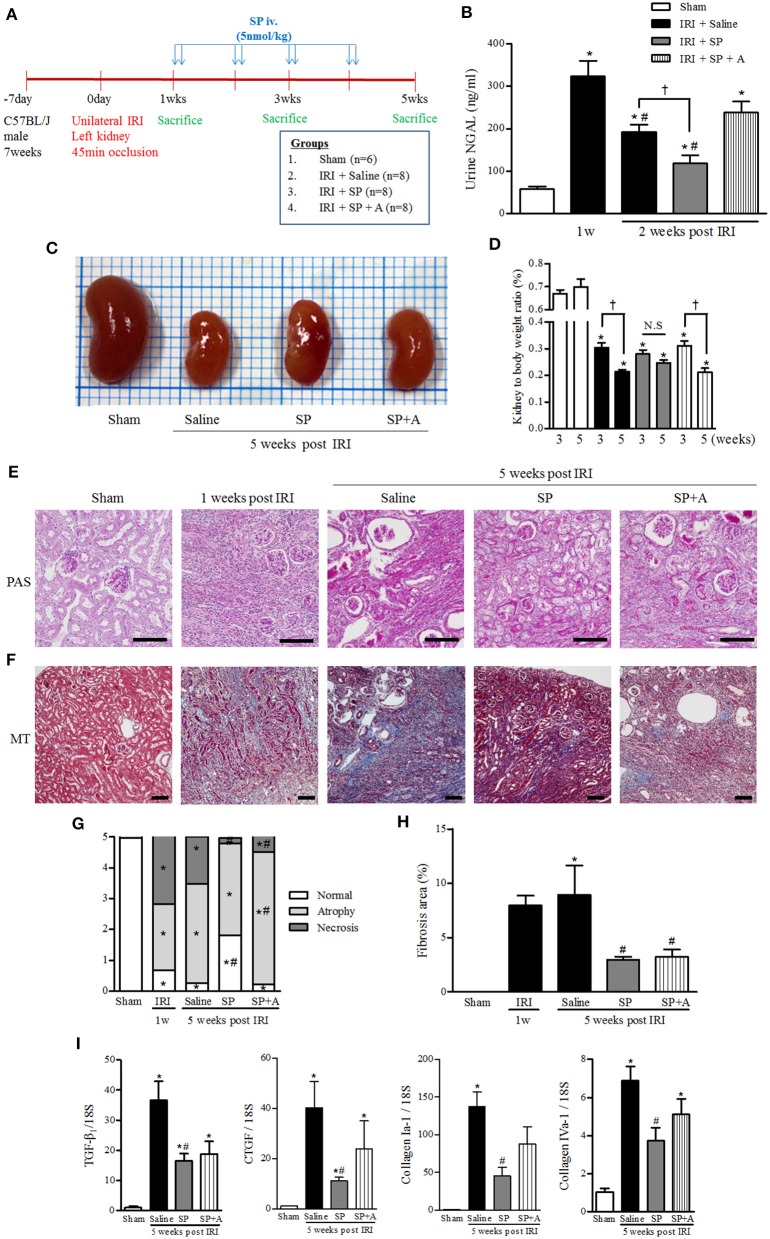
SP inhibited progressive renal injury and fibrosis after AKI. **(A)** Experimental design. Unilateral IRI was induced in the left kidneys of C57BL6 mice. After 1 week, SP was administered via the tail vein twice per week for 4 weeks. The mice were divided into the following four groups: normal control (sham, *n* = 6), saline treatment after IRI (IRI + saline, *n* = 8), SP treatment after IRI (IRI + SP, *n* = 8), and pretreatment with RP67580, an NK-1R antagonist, 15 min before SP treatment (IRI + SP + A, *n* = 8). **(B)** The concentration of NGAL in the urine was analyzed at 1 and 2 weeks after reperfusion. **(C)** Representative photograph showing the gross anatomy of kidneys at 5 weeks after reperfusion. **(D)** Changes in kidney weight to body weight ratio between 3 and 5 weeks after reperfusion. Values are shown as the mean ± S.E.M. ^*^p < 0.05 vs. the sham group, ^#^*p* < 0.05 vs. at 1 week after IRI group, ^†^*p* < 0.05 between the two indicated groups. **(E)** Periodic acid–Schiff (PAS) staining was used to detect kidney tubular injuries and to view necrosis, atrophy and normal tubules. **(F)** Masson's trichrome (MT) staining was used to detect kidney fibrosis (bottom) at 5 weeks after reperfusion. Scale bar = 80 μm. **(G)** Kidney tubular injuries were quantified by evaluating necrosis, atrophy and normal tubules based on a grading system that was used to produce scores. **(H)** Quantification of tubulointerstitial fibrosis. **(I)** The mRNA expression levels of fibrosis markers [from left: TGF- β1, connective tissue growth factor (CTGF), collagen I-a1, and collagen IV-a1] were determined using qRT-PCR. Values are shown as the mean ± S.E.M. ^*^p < 0.05 vs. the sham group, ^#^*p* < 0.05 vs. the IRI + saline group.

We hypothesized that SP, which modulates M2 polarization and bone marrow-derived cells after IRI, exerted a renoprotective effect against CKD progression after AKI. To support this hypothesis, we established a long-term unilateral IRI model and confirmed the SP effect by blocking the SP binding receptor (NK-1 receptor antagonist, RP67580, SP + A). At 1 week after IRI induction, SP was administered via the tail vein twice per week for 4 weeks (Figure 4A). Urine levels of neutrophil gelatinase-associated lipocalin (NGAL), a marker of tubular injury, were strongly increased at 1 week and moderately decreased at 2 weeks after IRI. Interestingly, urine NGAL levels in the SP-treated IRI group were significantly lower than the levels in the saline-treated IRI group at 2 weeks after IRI. Co-administration of RP67580, an NK-1 receptor antagonist, significantly reduced the effect of SP (Figure 4B). At 5 weeks after IRI, the kidney-to-body weight ratio was better preserved in the SP-treated IRI group than in the saline-treated IRI group (Figures 4C,D). One week after IRI, the most prominent histologic findings were changes in the necrotic tubules and massive infiltration of interstitial inflammatory cells. At 5 weeks after IRI, we observed a prominent progression to chronic injury, which is characterized by the cystic dilatation of Bowman's space, the sclerotic obliteration of glomeruli and the atrophic degeneration of most tubules. However, the SP-treated IRI kidneys exhibited significantly fewer necrotic tubules and higher numbers of normal tubular structures than the saline-treated IRI kidneys. The favorable effect that SP exerted on renal histology was abolished by treatment with RP67580. Normally shaped tubules were significantly restored in the SP-treated group, even compared with the histology at 1 week after IRI (Figures 4E,G). The area of interstitial fibrosis was also markedly smaller in the SP-treated IRI group than in the saline-treated IRI group at 5 weeks after IRI (Figures 4F,H). The mRNA levels of renal TGF-β1, CTGF, and type I and IV collagen were decreased in the SP-treated IRI group (Figure 4I).

### SP Reduced Renal Apoptosis After IRI

**Figure 5:**
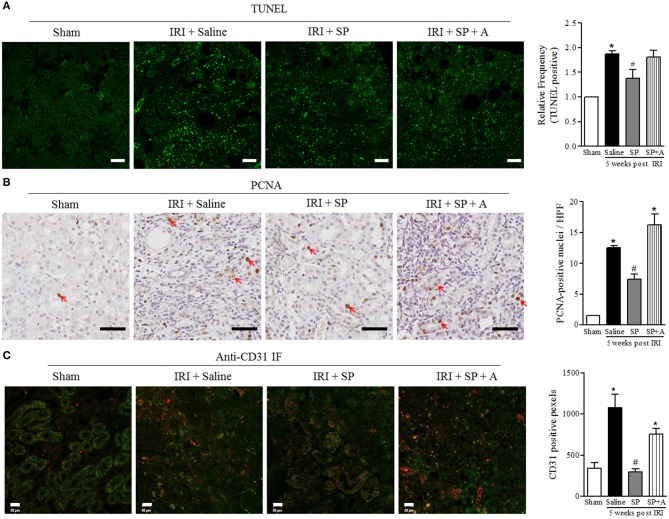
SP reduced renal apoptosis after IRI. **(A)** TUNEL staining and quantification of the frequency of TUNEL-positive cells in the kidneys at 5 weeks after reperfusion. **(B)** PCNA staining and quantification of the number of PCNA-positive nuclei (red arrow) in the kidneys at 5 weeks after reperfusion. **(C)** Immunofluorescence staining of CD31 and quantification of the peritubular capillary density, observed based on CD31 positivity (red fluorescence). Values are shown as the mean ± S.E.M. ^*^p < 0.05 vs. the sham group, ^#^*p* < 0.05 vs. the IRI + saline group. Scale bar = 20μm.

We analyzed the effect of SP on apoptosis and proliferation related to decreasing CKD progression. The number of apoptotic cells was lower in the SP-treated IRI group than in the saline-treated IRI group (Figure 5A). As shown in Figure 5B, SP-treated IRI kidneys displayed well-preserved tubular architecture and a decreased number of PCNA^+^ cells. These results indicated that SP prevents tubular cell death after IRI, and this effect was followed by a reduction in subsequent damage-associated proliferative activity. To determine whether SP affected the preservation of peritubular capillaries, we conducted immunofluorescence staining for CD31 in kidneys. As shown in Figure 5C, saline-treated IRI kidneys contained short, disorganized, tortuous peritubular CD31^+^ cells. The numbers of these cells were markedly increased in this group at 5 weeks but were approximately normal in the SP-treated groups. These results suggested that SP attenuates abnormal angiogenesis and stabilizes peritubular capillaries after IRI.

### SP Attenuated the Recruitment of Lymphocytes and Their Related Cytokines After IRI

**Figure 6:**
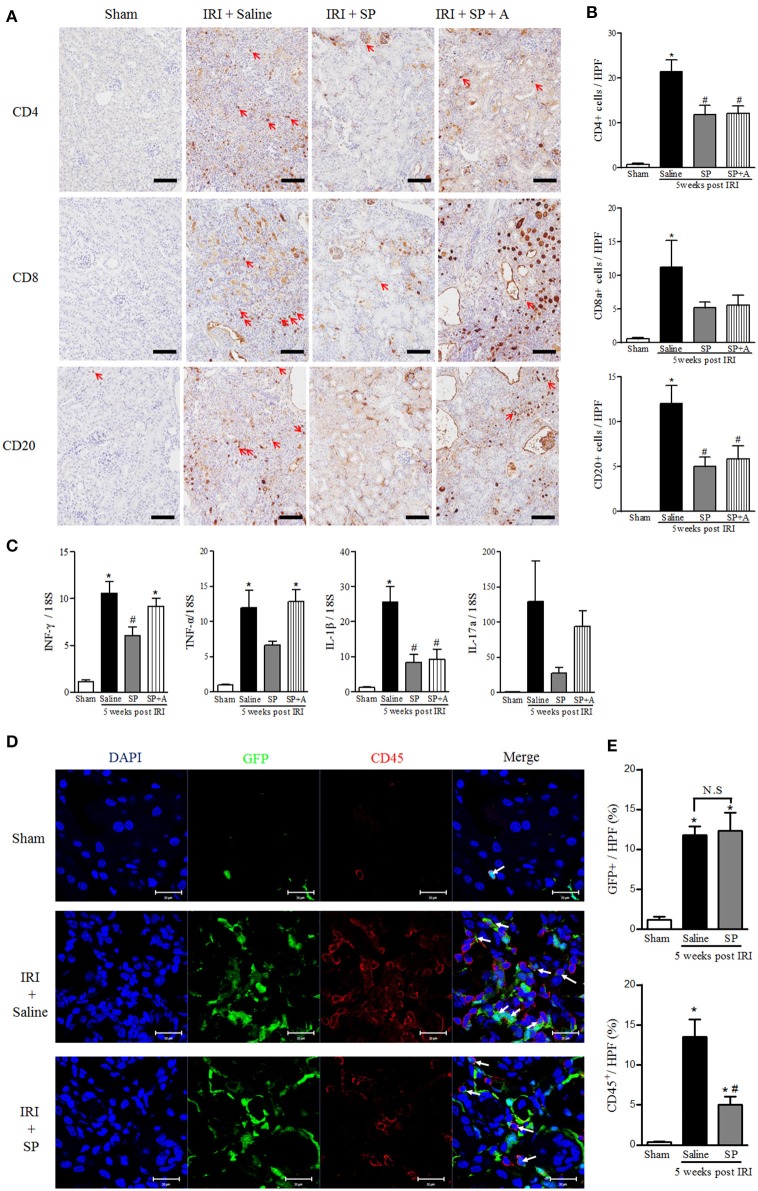
SP attenuated the recruitment of lymphocytes and their related cytokines after IRI. **(A)** Immunohistochemistry detection of CD4, CD8, and CD20. The positive cells are indicated by red arrows. Scale bar = 80 μm. **(B)** Quantification of the number of infiltrated CD4^+^ T cells (top), CD8^+^ T cells (middle) and CD20^+^ B cells (bottom) in whole kidneys. **(C)** The mRNA expression levels of macrophage- and inflammation-associated cytokine markers (from left, IFN-γ, TNF-α, IL-1β, IL-17a) determined via qRT-PCR. **(D)** Immunofluorescence was used to reveal bone marrow-derived GFP^+^ cells (green), CD45 (red), and nuclei with DAPI (blue). Scale bar = 20 μm. **(E)** The percent of the GFP^+^ and CD45^+^ area at 5 weeks after IRI. Values are the mean ± S.E.M. ^*^p < 0.05 vs. sham, ^#^*p* < 0.05 vs. IRI + saline.

To investigate the immunomodulatory effect of SP on macrophages and neutrophils, which influence adaptive immune cells at 5 weeks after IRI, we analyzed CD4^+^, CD8^+^, and CD20^+^ cells. As shown in Figures 6A,B, the numbers of infiltrated T and B cells were significantly lower in the SP-treated kidneys than in the saline-treated IRI kidneys. SP-treated kidneys also showed lower mRNA levels of the CD4^+^ T cell-related pro-inflammatory cytokines, such as IFN-γ, TNF-α, IL-1β, and IL-17a, than the saline-treated kidneys (Figure 6C). In the case of GFP^+^ bone marrow transplant chimeric mice, the number of GFP^+^ bone marrow-derived cells was increased after IRI, irrespective of treatment. Furthermore, the number of CD45^+^ cells was increased in the injured kidney. However, the increase in CD45^+^ cells was attenuated by SP treatment (Figures 6D,E).

## Discussion

SP is widely expressed in diverse cells, including epithelial cells, endothelial cells, immune cells, and MSCs (20, 21). Our study first described SP expressed in the kidney, especially tubular cells, and showed that it abruptly increased immediately after acute ischemic injury. Previous studies reported that SP regulates inflammation, wound healing, and tissue homeostasis through neurokinin 1 receptor (NK1R) (22). The effect of SP is different depending on disease or type of injury. For example, SP has been identified as one of the elimination targets since SP promoted the migration of monocytes and impaired natural killer (NK) cell cytotoxicity in HIV patients (23–25). Blockade of NK1R in DOCA-salt hypertension animal model, which increase plasma SP levels, reduced the kidney injury, fibrosis and macrophage infiltration (26). In neurogenic inflammation, SP is known as inflammatory mediator from brain and neurons (27). Furthermore, increasing severity of traumatic brain injury or ischemic time of stroke is correlation with increase of SP release and NK1R antagonist improve their pathological symptom (28, 29). Based on the above results, endogenous SP is one of the biomarkers associated with damage, and a rapid removal of SP, which transient increase after injury, appears to be a potential therapeutic target. Statins, which known as SP scavengers, decrease s SP and calcitonin gene-related peptide (CGRP) in sensory neuron and atorvastatin has protective effect on renal IRI (30, 31). However, the possibility, that the change of SP level is derived from damage reduction by statins and NK1R antagonist, cannot be ignored, because endogenous SP is injury inducible neurotransmitter and the protective effect of statin in kidney disease is associated with SP-independent inflammation and macrophage polarization (31, 32).

On the other hand, there are also a great deal of evidences that SP has a beneficial effect on wound healing and immune responses. Exogenous SP accelerated wound closure in full-thickness excisional wound healing model of non-diabetic and diabetic mice and the wound healing was delayed NK1R knock-out mice (33, 34). Proliferation and cell viability of retinal pigment epithelium (RPE) cells were enhanced by SP (35). SP also promoted RPE migration to prevent retinal degeneration in ARPE-19 cell and laser-induced retinal degeneration model (35). The generation of ROS and mitochondrial dysfunction under the diabetic environment were weakened by SP and it caused the epithelial wound healing (36). In the case of blood vessel regeneration, SP induced the recruitment of stem cells and the regeneration of smooth muscle cells, collagen layer and blood vessels in vascular graft explanted models (37). The angiogenesis and re-epithelialization in wound area was enhanced by SP with increase of ECM deposition (38). In the case of kidney disease, renal sensory responses related to SP and NK1R were impaired by hypertension and renal ischemia and administration of SP partially restored ARNA activity dose-dependent manner (39, 40). However, the beneficial effects of SP were not clear in kidney disease. In our study, SP clearly showed beneficial effects on acute renal injury and progression to fibrosis. IRI induced a pro-inflammatory response, tubular injury with necrosis, infiltration of CD4^+^, CD8^+^, and CD20^+^ cells and a long-lasting inflammation stage with abnormal proliferation and angiogenesis. SP treatment modulated the immune response, preserved normal tubular structures and attenuated interstitial inflammation and fibrosis even though we initiated SP treatment 7 days after IRI. These effects also disappeared after treatment with a NK1R antagonist, which inhibits a major receptor of SP. The mechanism of these beneficial effects of SP after IRI is associated with modulating macrophage polarization and bone marrow-derived cells.

At the early stage of AKI, various immune cells are activated and recruited in the injured kidneys (41). Both innate and adaptive immune systems contribute to the pathogenesis of tubulointerstitial inflammation and fibrosis and the process of wound repair (41). Macrophages are known as the critical regulator of inflammation and fibrosis (42). In addition, repression of pro-inflammatory cytokine from macrophage reduced the damage of AKI (43). For example, heat shock protein (HSP) 90 is associated with pro-inflammatory cytokine production of monocyte and macrophage and its inhibition reduced the damage of AKI (44, 45). Macrophages have two distinct phenotypes and functions, and their polarization is regulated by the surrounding microenvironment (46, 47). M1 macrophages are associated with cytotoxicity, anti-angiogenesis effects and fibrogenesis in kidney damage progression (48, 49). In contrast, M2 macrophages are associated with removal of necrotic cells, anti-inflammatory responses and regulation of the infiltration of lymphocytes in the proliferative phage of kidney repair (50, 51). The severity of renal injury in AKI and CKD is related to M1 macrophage infiltration, and its correlation was evaluated in diverse kidney diseases through depleting macrophage experiments (52). In particular, M1 and M2 macrophage polarization is important for regulating the balance between inflammation and tissue repair (46). Recent studies reported that SP induced M2 macrophages in the injured site by increasing the anti-inflammatory cytokine IL-10 in spinal cord injury (53, 54). Our study also showed that SP could induce the transition from M1 to M2 macrophages in the injured kidney. IRI induced CD11b^+^/CCR7^+^ M1 macrophage, CD68^+^/CD86^+^ dendritic cells and CD68^+^/CD169^+^ macrophages, which related to the activation of T and B cells (55). SP treatment decreased their populations and increased CD11b^+^/CD206^+^ or CD163^+^ M2 macrophages. However, one of the subtypes of M2 macrophages, M2a, is distributed in segmental glomerulosclerosis and tubular atrophy/interstitial fibrosis in IgA nephropathy patients and correlated with their severity (56). Interestingly, Lim et al. demonstrated that SP is an environmental factor that determines the polarization of macrophages and mediates SP-specific M2 polarization with M2c function but with M2a markers (19). Therefore, further studies will be needed on the role and subtypes of SP-induced specific M2 macrophages in diverse kidney diseases.

We also confirmed that SP modulated the bone marrow-derived cells after injury. Intriguingly, SP decreased bone marrow-derived neutrophils and increased the mobilization of MSCs to the kidney. The anti-inflammatory and immunomodulatory actions of MSCs induce the downregulation of the pro-inflammatory cytokines IFN-γ, TNF-α, and IL-1β and the upregulation of anti-inflammatory cytokines, such as IL-10 (57, 58). A recent report also showed that even inactivated MSCs could modulate monocyte function in response to LPS (59). Another study demonstrated that M2 macrophage polarization is induced by MSCs, which regulate the expression of cyclooxygenase 2 and indoleamine 2,3-dioxygenase (60). Regarding the relationships between SP and MSCs, SP activates the cellular response of MSCs, such as proliferation and migration, and blocks the senescence of MSCs (61, 62). Furthermore, SP restores the immunomodulatory function and number of MSCs in diabetic environments where stem cell function is reduced (63–65). The indirect immune regulation process of SP through MSCs may also be a mechanism that explains our results.

Because interstitial macrophage and lymphocyte infiltration is one of the reasons for progressive AKI, immunosuppressive drugs have been studied as an attractive therapeutic approach (66). Macrophage/monocyte depletion by clodronate reduced acute tubular necrosis at 1 day after IRI, attenuated inflammation and fibrosis and protected against functional loss for a long period after IRI (67, 68). However, cyclosporine and mycophenolate mofetil, an immunosuppressive agent that blocks initial leukocyte infiltration, aggravated morphologic damage and dysfunction after IRI (69). Rapamycin, which blocks the clonal expansion of T cells, worsened AKI damage by inducing the expression of heme oxygenase 1 and inhibiting mTOR-mediated proliferation of kidney cells (70, 71). Everolimus also attenuated the protective capacity of kidneys against oxidative stress by reducing heme oxygenase-1 expression (72, 73). Unlike conventional immunosuppressive drugs, SP can regulate the immune response and improve morphological changes after AKI. Administration of MSCs protects against kidney injury and functional loss by AKI through regulating the expression of inflammatory and oxidative markers (74, 75). However, stem cell therapy has critical obstacles to application in clinical settings, such as its side effects, high cost, and difficulty of use at the right time (76–78). Other strategies with better availability in clinical use than stem cells that have the similar effects and safety could replace stem cell therapy. Therefore, we focused on whether SP has immune regulation and stem cell mobilization capacity. Our results also suggested that SP treatment could be effective for the progression of CKD after AKI because we administered SP 7 days after IRI and attenuated tubulointerstitial fibrosis and inflammation. Given that AKI is due to various causes and has an unclear time point of injury initiation in clinical settings, this effect of SP in late AKI indicates its clinical availability as a treatment strategy for AKI.

In conclusion, we demonstrated that exogenous SP, which was recently defined as an injury-inducible messenger, mobilized bone marrow stem cells, downregulated M1 macrophages and neutrophils, induced M2 macrophage polarization, preserved normal renal tubular structures, attenuated renal inflammation and finally blocked the progression of kidney fibrosis after IRI. These favorable effects of SP in ischemic AKI should be investigated for candidate therapeutic target treatment for AKI.

## Data Availability Statement

All datasets generated for this study are included in the article/Supplementary Material.

## Ethics Statement

The animal study was reviewed and approved by Animal Research Ethics Committee of Kyung Hee University and Institutional Animal Care and Use committee Kyung Hee University Hospital at Gangdong.

## Author Contributions

D-JK and J-YM designed and performed all of the experiments. S-MK and J-WS performed and analyzed FACS. YL and SJ performed and analyzed confocal microscopy and light microscopy. KK, YK, and S-JL performed all of the histological analysis. SL designed unilateral IRI animal model and provided the method of animal model. D-JK, J-YM, S-HL, and YS wrote the manuscript. S-HL and YS coordinated the project. All authors participated in interpreting the results.

## Conflict of Interest

The authors declare that the research was conducted in the absence of any commercial or financial relationships that could be construed as a potential conflict of interest.
