# Supplementary material for: Substance P Improves Renal Ischemia Reperfusion Injury Through Modulating Immune Response
Source: Front Immunol. 2020 Apr 23;11:600. doi: 10.3389/fimmu.2020.00600 (PMC7190869; doi:10.3389/fimmu.2020.00600)
Supplement: Supplementary file 1 [file Data_Sheet_1.PDF]

frontiers

Supplementary Material

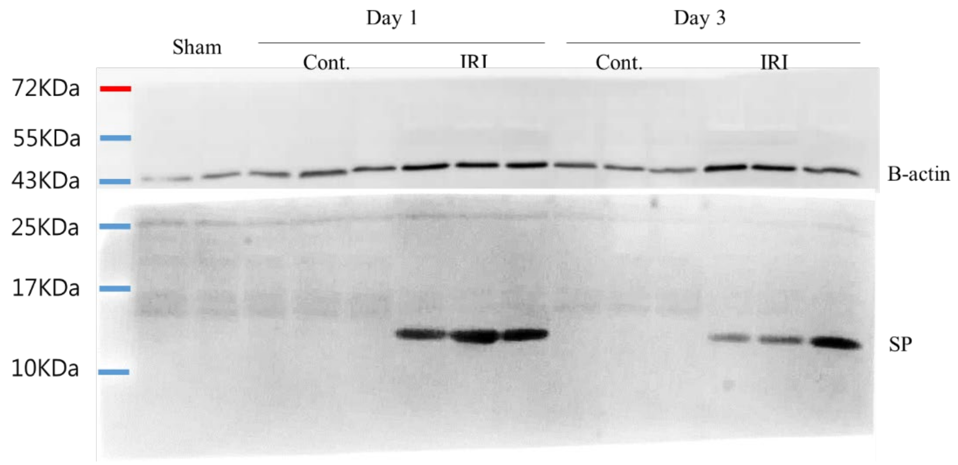

Supplementary Figure 1. The original image of Fig 1B.

Gene	Sequence		Gene	Sequence	
TGF-β_1	(Forward)	5'-CAACAATTCCTGGCGTTACCTTGG-3'	CTGF	(Forward)	5'-GAGTGTGCACTGCCAAAGAT-3'
	(Reverse)	5'-GAAAGCCCTGTATTCCGTCTCCTT-3'		(Reverse)	5'-GGCAAGTGCATTGGTATTTG-3'
Collagen Ia-1	(Forward)	5'-CGACCTCAAGATGTGCCACT-3'	Collagen IVa-1	(Forward)	5'-CCTTCATTAGCAGGTGTGCG-3'
	(Reverse)	5'-CCATCGGTCATGCTCTCTCC-3'		(Reverse)	5'-CGATCCACAGTGAGGACCAA-3'
VEGF-A	(Forward)	5'-TTCGGGAACCAGACCTCTCA -3'	VEGFR2	(Forward)	5'-TTTGGCAAATACAACCCTTCAGA-3'
	(Reverse)	5'-GACCCAAAGTGCTCCTCGAA -3'		(Reverse)	5'-GCAGAAGATACTGTCACCACC-3'
Angiopoietin-1	(Forward)	5'-CACATAGGGTGCAGCAACCA-3'	Angiopoietin-2	(Forward)	5'-CCTCGACTACGACTCAGT-3'
	(Reverse)	5'-CGTCGTGTTCTGGAAGAATGA-3'		(Reverse)	5'-TCTGCACCACATTCTGTTGGA-3'
INF- γ	(Forward)	5'-CGGCACAGTCATTGAAAGCC-3'	TNF-α	(Forward)	5'-GTGCCTATGTCTCAGCCTCT-3'
	(Reverse)	5'-TGCATCCTTTTTCGCCTTGC-3'		(Reverse)	5'-CTGATGAGAGGGAGGCCATT-3'
IL-1β	(Forward)	5'-ACAACTGCACTACAGGCTCC-3'	IL-17a	(Forward)	5'-ACTCTCCACCGCAATGAAGA-3'
	(Reverse)	5'-GCTTGGGATCCACACTCTCC-3'		(Reverse)	5'-CTCTCAGGCTCCCTCTTCAG-3'
18S	(Forward)	5'-GTAACCCGTTGAACCCCATT-3'	TAC-1	(Forward)	5'-GGTCTGACCGCAAAATCGAA-3'
	(Reverse)	5'-CCATCCAATCGGTAGTAGCG-3'		(Reverse)	5'-TCGGACCAGTCGGACCAATA-3'

Supplementary table 1. Primer sequence for qRT-PCR

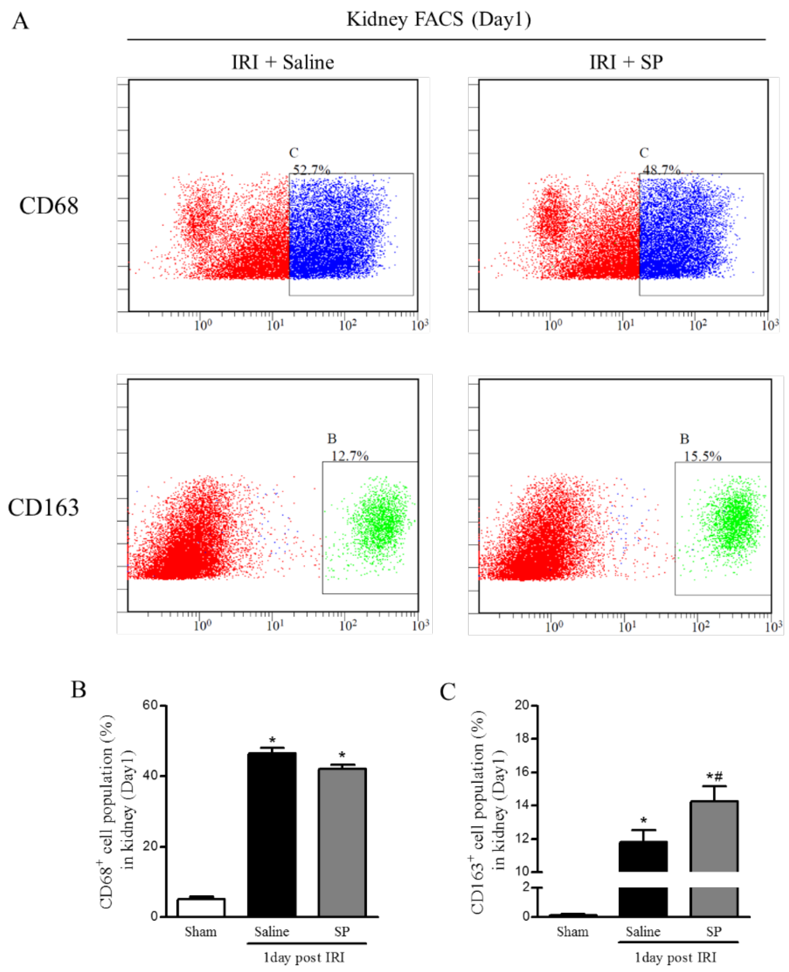

Supplementary Figure 2. SP modulated IRI-induced macrophage polarization. Unilateral IRI was induced in the left kidney in C57BL6 mice. Saline or SP was administered to mice at 1 day before IRI and immediately after reperfusion. The mice were sacrificed at 1 day after IRI. (A) The intrarenal $CD68^+$ and $CD163^+$ cell populations. (B) The percentage of intrarenal $CD68^+$ cell populations. (C) The percentage of intrarenal $CD163^+$ cell populations. Values are shown as the mean±S.E.M. *p<0.05 versus the sham group, $^{\dagger}p$<0.05 versus the contralateral kidney, #p<0.05 versus the IRI+saline group.

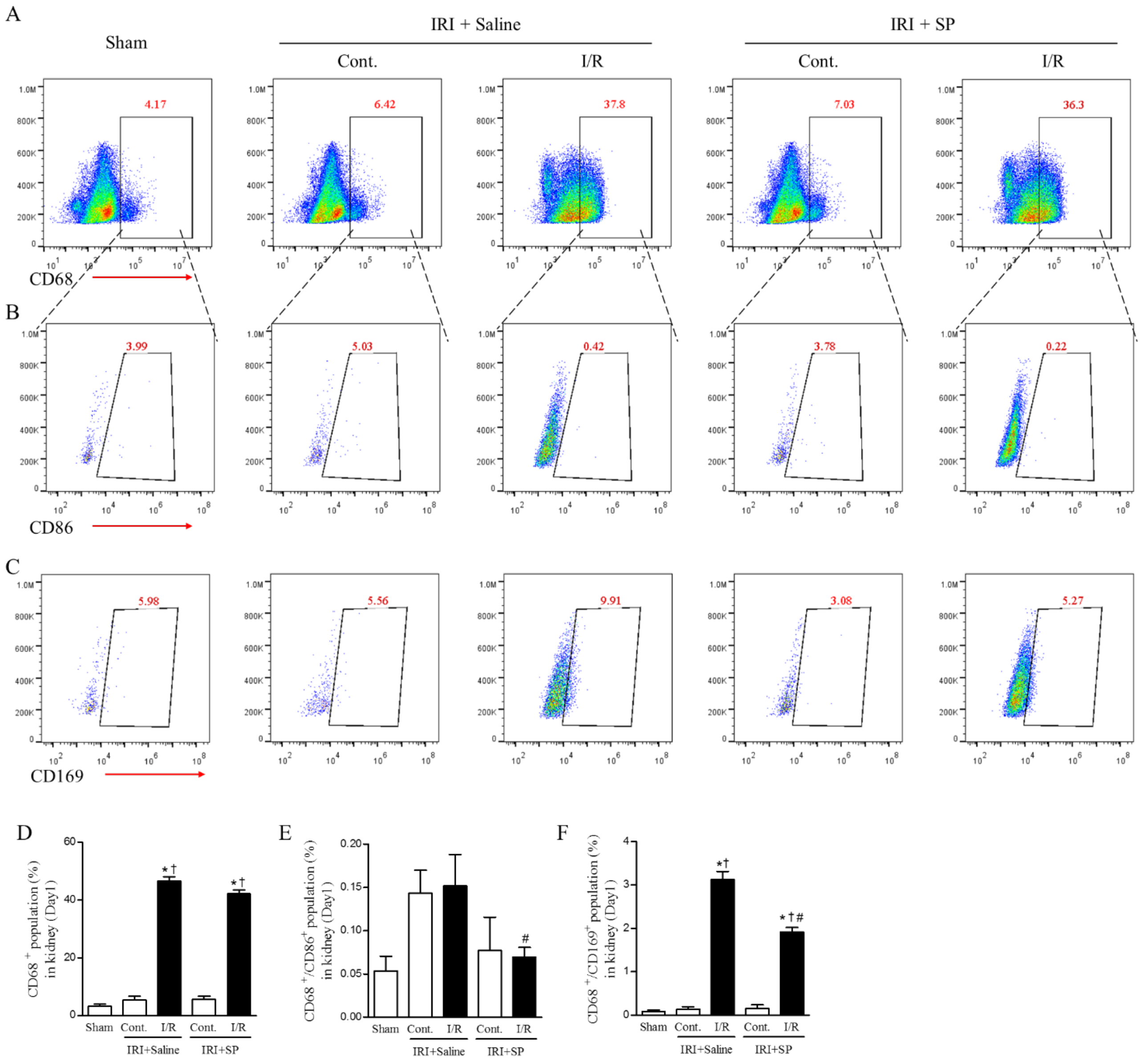

Supplementary Figure 3. SP modulated IRI-induced macrophage polarization. (A) The intrarenal $CD68^+$ cell populations. (B) The intrarenal $CD68^+/CD86^+$ cell populations. (C) The intrarenal $CD68^+/CD169^+$ cell populations. (D) The percentage of intrarenal $CD68^+$ cell populations. (E) The percentage of intrarenal $68^+/CD86^+$ cell populations. (F) The percentage of intrarenal $CD68^+/CD169^+$ cell populations. Values are shown as the mean±S.E.M. **p*<0.05 versus the sham group, $^{\dagger}p$<0.05 versus the contralateral kidney, #*p*<0.05 versus the IRI+saline group.
